# On the Masturbation, Spermatorrhœa and Impotence of Adolescence

**Published:** 1879-05

**Authors:** James Nevins Hyde


CHICAGO MEDICAL

JOURNAL & EXAMINER.

VOL. XXXVIII.—MAY, 1879.—No. 5.

[In these pages dimension and weight are expressed in terms of the Metric System, and temperature in degrees of the Centigrade Scale.]

### Original Lectures.

ARTICLE I.

### ON THE MASTURBATION, SPERMATORRHŒA AND IMPOTENCE OF ADOLESCENCE. A lecture delivered at Rush Medical College, April 10, 1879, by JAMES NEVINS HYDE.

GENTLEMEN :—Few disorders occasion greater mental distress to the patient than those, real or fancied, which are referred to the male organs of generation. Few also present more perplexing problems to the consideration of the physician. So intimate is the relation between the brain and the sexual organs, that a mere impression upon the eye, or an emotion, is sufficient in many men to produce turgescence of the penis. In case, therefore, of a real or fancied disease of these parts, the trouble is largely originated, aggravated or corrected by the mental condition.

It is especially with this important fact in view, that I desire to-day to consider those aberrations of adolescence, which are betrayed in masturbation and the conditions commonly described as spermatorrhœa and impotence.

The subject is a momentous one, and has naturally not failed of due consideration by competent authors. Still it has been neglected or lightly treated by many writers, for the reason, it may be supposed, that the theme has been especially chosen for the exploitations of the unscrupulous charlatans, whose ignorant and pretentious pamphlets are written and circulated for the purpose of enriching their authors at the expense of the timid. Again, our medical periodical literature, of late years especially, has been concerned chiefly with the medical and surgical treatment of cases of this class. But the necessary and useful measures suggested, seem to me to bear no comparison in importance with what may be called the rational treatment of the mental condition of these patients.

The period of pubescence in the male, no less than in the female, is attended with marked and significant phenomena. In the case of many young men it lapses without producing physical disturbance, as some children escape dentition without serious annoyance. With other youths it is different. Ignorant of the physiology of the sexual organs, or instructed only from the lips of their misinformed companions, the light which they can throw upon the subject is usually of a very distorted and discolored quality. In the midst of the social surroundings of the present day, it cannot be questioned that there is frequently a precocious awakening of the sexual instinct. Whether, however, that awakening be precocious, tardy or timely, it is tolerably sure to make itself known. The genitals become physiologically congested, the seminal vesicles distended with their contents, the testicles turgid, and the erections of the penis, habitual with every healthy lad, become less frequently the reflex evidence of a full bladder or a distended bowel, and more often the sign and symptom of a true sexual impulse. Every one knows that these are the natural preparations for the reproduction of the race.

Now if it were possible to seize this moment in each instance, it would be a wise and timely course to then instruct the boy with regard to his awakening powers. He should be told that every healthy man has sexual instincts, that no man need be ashamed of such instincts; that he only need feel ashamed of such instincts who permits them to control him or to entice him into depraved practices; that, properly controlled, his desires will eventually be the source of an actual benefit to him in mind and body ; and that, if improperly controlled, they may produce a great deal of wretchedness and misery.

Unfortunately, such a course is rarely possible. In a certain proportion of cases, masturbation is begun either under the teachings of others of his own age, or solely from the promptings of the period of his life.

Certainly at this day, it is unnecessary to repeat that the sequence of events we have considered, is often precipitated by the physical conformation of the lad. I have heretofore dwelt upon the fact, that a congenitally tight or redundant prepuce may lie at the foundation of a large proportion of these cases. If the mucous surface of the glans be kept in an exquisitely sensitive state by its tight envelope, if it be further irritated by an imprisoned smegma, it can be well understood how the manipulation of the parts in the act of preparing to urinate, and even the accidental contacts of the sled, the back of a pony, the tree up which he climbs, and even the bedclothing at night, are capable of awakening in the boy at once a revelation and a revolution. Every father should be advised to see that his son has a fair chance in the struggle of life, where the fittest survive, guaranteeing to him a free prepuce, and a clean glans. None the less, should it be certain that a hernia is properly supported, a varicocele attended to, and a tendency to prolapse of the rectum relieved. These and possibly a few other morbid conditions are capable of doing mischief in consequence of the reflex irritation they occasion.

Now it happens in a large number of cases, that these young masturbators sooner or later become alarmed at their practices, in consequence of some information they receive. Often this latter is of a most mischievous character. Occasionally too, the religious element is predominant, and the mental condition of these young men becomes truly pitiable, even when at intervals, impelled by a misguided passion, they continue to handle themselves. Some are overcome and entirely unmanned by the "sins" they have committed, and curse themselves bitterly in their moments of depression. Others believe that they have accomplished the ruin of body and soul, and are already condemned for their misdeeds, to a life of misery in this world, and a future of despair in the next. This is no overdrawn picture. I have received letters on this subject, that are eloquent enough to excite the sympathy of the most unimpressible; and am satisfied that a certain small proportion of unexplained suicides, are the result of this mental strain. A full and free confession to one who is wiser than they in these matters, is of immense value to these unfortunates. There is a burst of sobs and tears; and the whole trouble is often over after they have received some sound information and advice.

The facts are nearly these: Masturbation is not a crime nor a sin, but a vice. They who make confession with promise of reform, should be kindly told the truth about it. Though a vice merely, it is nevertheless a very filthy and disgusting vice, and should be classed with such degrading habits as picking the nose and scratching the anus. The very worst that masturbation does for the immense majority of those who practice it, is the loss of self respect it occasions, and the despondent mental condition it often brings to those who become morbid with respect to its injurious consequences. The best thing to say to these patients is to assure them that they have already reaped the fruit of their folly in their misery; that no possible evil consequences will follow in the future so long as they amend; and that the best way to treat the habit of masturbation is to abandon and forget it, as they would any other nastiness.

I say that no evil consequences will follow, for such certainly is the experience of the best authors upon the subject. Sir James Paget remarks, in his clinical lecture upon Sexual Hypochondriasis: "I believe you may teach positively that masturbation does neither more nor less harm than sexual intercourse practiced with the same frequency in the same conditions of general health and age and circumstances."* This admirable essay is classical in the field which we are now surveying, and I know of no other upon the same subject of equal value. The author goes on to show that when there is mischief, it results rather from the quantity than the method of the excesses; and that he had never seen nor heard anything which would lead him to believe that the ef-

* Clinical lectures and essays, London. Longmans, Green & Co., 1875, p. 284.

fects of occasional masturbation were different from those of intercourse, or that there was any justification for the special dread with which many sexual hypochondriacs regard it.

Dr. G. M. Beard pertinently remarks* that the genital organs were designed to bear a vast amount of abuse without permanent injury, and, respecting the relative harmfulness of the natural and unnatural methods of excitation, he thinks masturbation is worse simply because it can be practiced earlier in life, also at any time when alone, and therefore more frequently. Dr. H. C. Wood† expresses his belief as follows: "Some persons think that masturbation is more injurious in its effects than excessive venery. I do not believe this."

My own convictions upon this point are positive. During a part of my professional career I was the surgeon of a man-of-war, and had the opportunity of carefully observing and treating for long periods of time, hundreds of men, varying in age from that of the messenger-boy to the veteran of the top, some of them from the lowest and worst ranks of life, living between the decks of a vessel, completely cut off from the companionship of females for months at a time, many of them indulging their appetites without restraint in more than one unnatural manner. Since the years of the war, I have had the opportunity of carefully watching a large number of men of another class — those who, possessed of means and opportunities, have indulged in excessive sexual intercourse, both within and without the pale of matrimony. There cannot be a question but that the last named have been by far the worse sufferers. Miserable as must be the unnatural gratification of the appetite, we must admit that excesses of a natural sort are worse in their remote consequences.

For, let it be noted, that in the immense majority of cases, even after years of indulgence, sooner or later masturbators abandon their habit. Fear, courage, marriage, the wider knowledge and self-restraint that come with years—these, and a host of other influences operate finally with most men to end their trouble. They became husbands and fathers, and it may be presumed, their lives are the sweeter for the conflict they have sus-

* Proceedings of the Medical Society of the County of Kings, April, 1878.

† Clinical Lectures, Phil. Medical and Surgical Reporter, Dec. 22, 1877; p. 481.

tained. Our countryman, Hawthorne, has drawn a true picture of this conflict, in his criticism of the famous painting by Guido, in the Church of the Cappuccini, in Rome, representing Michael, poised in the flush and triumph of victory, with one foot upon the neck of the prostrate demon. Not even an archangel, writes Hawthorne, could escape from such a struggle with unruffled feathers.

Naturally enough, soon after he abandons his practices, the young man will notice that he has nocturnal discharges of semen, sometimes with, sometimes without dream or erection. If he were anxious when he had not abandoned the habit, his anxiety is now increased ten-fold. He peruses some wretched pamphlet on "Lost Manhood," only to find there his worst fears confirmed. He has sowed the wind, surely he is now to reap the whirlwind. Very probably, too, the extrusion at intervals of a mass of hardened fæces from the rectum, has squeezed a small quantity of the prostatic fluid from his urethra, which is to him conclusive evidence that he is the victim of "spermatorrhœa." If he can find also a few shreds of mucus from the prostatic urethra in the urine, or can discern, after any occurrences of a kind calculated to stimulate the genitals, a transparent fluid escaping from the urethra, the cup of his misery is nearly full.

But what are the facts? The young man who has masturbated has probably succeeded in simply awakening and developing prematurely his sexual powers. If he had never masturbated, the chances are that, sooner or later, he would have observed that he had nocturnal discharges. I never knew of a chaste celibate leading a sedentary life, who did not at times, during the years of sexual power, have similar annoyances. The passage from the urethra of a small quantity of prostatic fluid or the secretion of Cowper's glands, either during expulsive straining at stool or, obviously as a preparatory lubrication, after stimulation of various kinds, is common enough.

None of these is a symptom of disease—certainly not of true spermatorrhœa. Perhaps it would be well to declare to you here and now the fact of the great rarity of true spermatorrhœa. I feel quite sure that more young men suffer annually from hydrophobia and the stroke of lightning, than from this bugbear of timid and nervous youths, and yet the mass of mankind spend their days without apprehension of mad dogs or thunder storms.

True spermatorrhœa is a disease rather of middle life and advanced years. It is significant, generally, of a grave disorder of the spinal medulla. An unprovoked discharge of semen in the daytime is far more rare than four-leaved clover. Even the nocturnal discharges of youth are largely made up of the liquor prostatæ.

Fluids of this character are secreted to be excreted. The mistake of many lies in assuming that they are secreted to be stored for use at some future crisis. But the testis is for secretion, and there is no storeroom of the genitals larger than the seminal vesicle. A seminal vesicle distends with its contents only that it may be relieved of them. It is probable that in a state of celibacy, a certain number of nocturnal discharges each month are positively beneficial. For a day or two days after this event there may be an unusual degree of languor and a sense of fatigue, but the tone of the system is afterward rapidly restored.

The analogy in the female is very perfect. The periodical emissions from the unimpregnated uterus. correspond very fully with the periodical discharges from the prostate, which is conceded to be the uterine analogue. In each case, the discharge may or may not be associated with the ejection of spermatozoa or ova. It is strange that young women, often those hysterically inclined, who have once experienced the alarm of a first menstruation, can regard thereafter with composure a periodical occurrence, the like of which, in the case of the young man, may become to him the source of a profound dejection. Nor does the analogy between the two cease at puberty. It is when nearing the period of the menopause that we look in women for that sub-peritoneal, sub-mucous or interstitial uterine fibroid development which in men of fifty or sixty years of age is represented in prostatic hypertrophy and interstitial or polypoid tumors of precisely analogous texture.

But, it may be objected, there is no marked periodicity in the expulsive function of the prostate. This is unquestionably an error. A periodicity certainly exists, though it may be, and often is, masked by the stimuli of the imagination and of modern social life in large cities. Married men have often admitted to me the fact that, at certain periods of the month, they were very much more disposed to sexual indulgence than at others, and have been immensely surprised to learn that in this particular they were by no means singular. The same thing is probably true of the unmarried youth who is not unduly stimulated. There are periods in the month when the prostate is more disposed to functional activity than at others. Hence it is that, at times, a young man will have discharges several times during the same night, or even several nights in succession. The fact is to him portentously alarming; and might be indeed, if such a state of affairs were permanent. But such a state of affairs never is permanent. Nature is more conservative than the apprehensions of puberty. There is a prostatic no less than a uterine ebb ; and these periods of prostatic activity will be succeeded by periods of prostatic repose, if only the youth will dismiss his anxieties and fears, and suffer his generative organs to repose, where they were intended to repose, under the shadow of the laws which govern the involuntary or, as Bichât calls it, the vegetative half of his organism.

But, unless instructed, he will rarely do this. He approaches his physician with complaints that have the exact flavor of the advertisements of irresponsible men in the daily newspapers, and the effect would be ludicrous were the complainant not seriously alarmed about himself. He is afraid of insanity. But the truth is that only a very small number of persons who live in apprehension of insanity, ever become insane. The one characteristic of insidious cerebral disease is the unconsciousness of the patient that he is losing, or has lost, his mental equipoise. The man of whom the skilled physician becomes suspicious, is he who thinks it necessary to insist upon his sanity. Many insane males masturbate, and I have seen insane patients with singular delusions respecting the subject of their masturbation and their sexual organs, but these are assuredly the results rather than the causes of the mental derangement.

The young man whose case we are considering, will often complain also that he is losing his memory, and that he sees "spots dancing before his eyes." But the power of memory is the result largely of education. The memory of many insane patients is in the highest degree excellent, and some of the healthiest and most intelligent men whom you ever meet, are so deficient in memory and absent-minded, that they are the laughing stock of their acquaintances. Every physician knows furthermore that slight corneal opacities, floating particles in the humors of the eye, and even retinal derangements, may produce images of spots—the muscæ volitantes of writers—which are consistent with perfect health.

I have said that the sedentary life of the youth in our large cities, is responsible for a great share of this sexual hypochondriasis. Hundreds of thousands of young men in this land were tormented in a similar way eighteen years ago, when the turmoil of civil war first agitated the country. They abandoned their sedentary lives, entered camp, lived on the coarsest fare, struggled with musket and knapsack over their long marches, and sank down wearily at night, often on the bare ground, into the most blessed and dreamless repose. To their astonishment they forgot, because they were compelled to forget, their sexual complaints. In this way the great war proved a blessing to whole regiments of young men.

But the last and sorest complaint of the youths of this class, is that they are "impotent." The question of matrimony is presented to them finally, and they recoil before it as before a plunge into a cold bath. Many of them will say that they have tested themselves, and found that they were utterly incapable of effecting intercourse. Upon the slightest contact with the female genitals they have, they report, a seminal discharge, even before the possibility of the occurrence of an erection. Or, if an erection has occurred, it was so feeble and transitory, that almost immediately after the intromission of the organ, the ejaculation was accomplished. Perhaps they will confess that they were rallied or reproached by their companion for their failure, and that they have been brooding over this misfortune till the burden has become will nigh intolerable.

Now this question is one of importance. The great, the pressing need of many of these young gentlemen, is a proper sexual hygiene. They need physiological rest of the sexual organs, not absolute rest. Absolute rest is the doom of the Oriental eunuch. Physiological rest is the boon of regular and temperate gratification of desire. A promise of this rest is held out by marriage; and that proffered by any illegitimate relation, is pregnant with disaster and danger. How can we ensure for them this physiological rest, and in what cases can we advise marriage with confidence?

It seems to me that there is great and general misunderstanding about this entire question of virility. 'Tis a shame that such an honest word should come to have its splendid quality apply only to that baser office in man, which he shares with his first cousins of the brute creation.

The sexual act is by no means the supreme expression of manhood. Celibacy in both sexes is compatible with the attainment of the highest types of physical excellence. I have not the time, nor is this the place, for a digression in which to describe those nobler gifts of a true virility which have, in all ages, distinguished the best and worthiest of our sex. We must be content with pointing to the fact that the idiot, the imbecile, the consumptive, the syphilitic and even the paralytic of the male sex, have again and again reproduced their kind, and are to-day burdening every commonwealth with the care of their ill-begotten offspring. But to the youth who is inflamed with passion, the act assumes an immense and undeserved significance. The whole heavens are brass till his ardor is quenched. Well were it for him and for those like him, if they could express the sentiments of that rare and wise physician Sir Thomas Browne:

"I could be content that we might procreate like trees, without conjunction, or that there were any way to perpetuate the world without this trivial and vulgar way of coition; it is the foolishest act a wise man ever commits in all his life; nor is there anything that will more deject his cooled imagination, when he shall consider what an odd and unworthy piece of folly he hath committed."*

Turgescence, erection, and intromission of the male organ are not essential to procreation. The one requisite is the supply of the

* Relig. Medici, Ticknor and Fields, Boston, 1862, p. 137.

male element; and true aspermatism, when double epididymitis has not preceded, is rare indeed. It is at times merely necessary that the spermatic fluid should be applied over the external genitalia of the female, to insure impregnation. Even when there is diurnal ovulation, as in the fowl, the application of the ano-genital orifice of the cock to the corresponding part of the hen, will insure fertility. The records of medicine are full of illustrations of the fact that an impenetrable hymen, spasm of the sphincter vaginæ, and even amputation of the penis, constitute no bar to pregnancy.

But the young man who pronounces himself "impotent," need not be dismissed to this category in the list of generators.

Unquestionably men differ in their sexual powers just as they differ in the shape of their noses or the length of their ears. There are those of such unusual vigor that they can perform the prodigious feats of which Trousseau writes in his Clinical Lectures. Such men are capable of committing rape at will upon an indefinite number of women, and it would seem that the males of certain tribes of our North American Indians possess a power of this kind. At the other extreme we find the men of a nervous and peculiarly emotional type. By these the sexual act can be performed only with those women to whom they are peculiarly susceptible. For all others they might be called impotent. A mental emotion, a word, the sight of an offensive object, the recollection of a disagreeable occurrence, instantly renders them temporarily incapable of action, even in the presence of those with whom they have sustained relations.

Between these two extremes are to be found the mass of men—among them the nervous and despairing youths who have been for long bemoaning their lost manhood. For them, a certain degree of education is requisite before the act can be properly accomplished. In their illicit approaches to women, the very novelty of their situation has unhorsed them. Unaccustomed to the act, the person, the organs, stimulated in the highest degree by anticipation of what is to follow, their seminal vesicles usually distended to the utmost with a secretion perhaps long pent-up, the prostate lies ready on the instant to discharge its burden.

The wonder is, not that they fail in such intercourse, but that they should, as they sometimes do, succeed.

Now the married state offers just the proper school for an education of this sort, and it should be recommended with confidence to every man who is capable of supporting a wife and family ; who is capable of secreting and ejecting semen from the external meatus of his urethra, and who, once or twice in the week or month, arises from sleep to find his penis in a state of vigorous erection. Such a man will plead that he is sure to fail, and the proper response should be always—

"Of course you will fail. A very considerable proportion of bridegrooms fail in their first marital approaches, and you cannot expect to escape such a common lot. You will fail the first time and the second time, and possibly many times more for a few weeks. But what of it ? The failure will prove, in all probability, to be a mutual one. If you have married an innocent and virtuous as well as an affectionate girl, and you ought to marry no other, she will be as ignorant of these matters as I am confident you are. The difficulties which beset your way in this direction, will none the less beset hers. And so you will go on, both of you, correcting mistake after mistake, and error after error, till the undue excitement and the novelty of the situation shall have worn away. Then will arrive a supreme moment when the act will be perfectly consummated ; and so long thereafter as you are true to the one woman with whom you cohabit, your resources will never fail. The whole burden of your sexual trouble will be lifted away from your life like a dark cloud, and with it you will do well to drive away from your memory the recollection of the filth and nastiness which unfortunately pollute too often the springs of a normal adolescence."

A man, married six months or more, may give a history of masturbation and nocturnal emissions before marriage, and also complain that, since then, he has been totally unable to perform the sexual act properly. But the number of those men, in whom no positive and present cause of their trouble can be detected, is so small as to be insignificant. I remember the case of a gentleman who gave a history of this kind, and who positively declared that, during the five years of his married life his erections had always been imperfect, and intromission instantly succeeded by ejaculation. He was well nourished, happy in his domestic relations, and not in the least worried about his condition. He had a healthy child, whose existence, he said, was a standing surprise to him. He complained of other symptoms, and for these I explored his urethra. I found a tight stricture one inch from the meatus. This I divided. During the subsequent treatment his wife conceived a second time, and he told me that he had effected natural intercourse at the date of this conception.

It may be said, in general, that the married men who complain of "impotency" are almost invariably those in whom this condition has succeeded to the previous enjoyment of their powers. But these do not belong to the class of adolescents, whom we are now describing.

I would not have you infer from what I have said, that medical and surgical treatment is not needed by many of these complainants. It is indeed essential to the judicious management of a certain proportion of cases, and I will endeavor to treat this part of the subject in a future lecture, deferring to that time a consideration of the effect upon the genital organs of the habitual use of tobacco, alcoholic stimulants, and opium.

